# Microbial Diversity and Metabolic Potential in the Stratified Sansha Yongle Blue Hole in the South China Sea

**DOI:** 10.1038/s41598-020-62411-2

**Published:** 2020-04-06

**Authors:** Peiqing He, Linping Xie, Xuelei Zhang, Jiang Li, Xuezheng Lin, Xinming Pu, Chao Yuan, Ziwen Tian, Jie Li

**Affiliations:** 1 Key Laboratory of Science and Technology for Marine Ecology and Environment, First Institute of Oceanography, Ministry of Natural Resources, 6 Xianxialing Road, Qingdao, 266061 China; 2 Laboratory for Marine Ecology and Environmental Science, Qingdao National Laboratory for Marine Science and Technology, Qingdao, 266071 China; 3 Key Laboratory of Natural Products of Qingdao, Qingdao, 266061 China; 4 Research Center for Islands and Coastal Zone, First Institute of Oceanography, Ministry of Natural Resources, 6 Xianxialing Road, Qingdao, 266061 China; 5 Marine Engineering Environment and Geomatic Center, First Institute of Oceanography, Ministry of Natural Resources, 6 Xianxialing Road, Qingdao, 266061 China

**Keywords:** Water microbiology, Microbial ecology

## Abstract

The Sansha Yongle Blue Hole is the world’s deepest (301 m) underwater cave and has a sharp redox gradient, with oligotrophic, anoxic, and sulfidic bottom seawater. In order to discover the microbial communities and their special biogeochemical pathways in the blue hole, we analyzed the 16S ribosomal RNA amplicons and metagenomes of microbials from seawater depths with prominent physical, chemical, and biological features. Redundancy analysis showed that dissolved oxygen was the most important factor affecting the microbial assemblages of the blue hole and surrounding open sea waters, and significantly explained 44.7% of the total variation, followed by silicate, temperature, sulfide, ammonium, methane, nitrous oxide, nitrate, dissolved organic carbon, salinity, particulate organic carbon, and chlorophyll *a*. We identified a bloom of *Alteromonas* (34.9%) at the primary nitrite maximum occurring in close proximity to the chlorophyll *a* peak in the blue hole. Genomic potential for nitrate reduction of *Alteromonas* might contribute to this maximum under oxygen decrease. Genes that would allow for aerobic ammonium oxidation, complete denitrification, and sulfur-oxidization were enriched at nitrate/nitrite-sulfide transition zone (90 and 100 m) of the blue hole, but not anammox pathways. Moreover, γ-Proteobacterial clade SUP05, ε-Proteobacterial genera *Sulfurimonas* and *Arcobacter*, and Chlorobi harbored genes for sulfur-driven denitrification process that mediated nitrogen loss and sulfide removal. In the anoxic bottom seawater (100-300 m), high levels of sulfate reducers and dissimilatory sulfite reductase gene (*dsrA*) potentially created a sulfidic zone of ~200 m thickness. Our findings suggest that in the oligotrophic Sansha Yongle Blue Hole, O_2_ deficiency promotes nitrogen- and sulfur-cycling processes mediated by metabolically versatile microbials.

## Introduction

O_2_-deficient regions occur throughout global oceans^1^. Intermediate layers of the ocean develop O_2_-deficient water masses, referred to as oxygen minimum zones (OMZs), due to limitation in photosynthetic O_2_ production and high-level aerobic respiration during the degradation of surface-derived organics^2^. In these OMZs, such as the Eastern Tropical South Pacific (ETSP) and the Arabian Sea, O_2_ concentrations fall below sensor-specific detection limits^3–5^. In contrast, the Peru Upwelling Region, the Namibian Shelf, and the Indian Continental Shelf experience episodic plumes of hydrogen sulfide (H_2_S)^6–8^. These sulfidic environments are also found in enclosed or semi-enclosed basins, including the Black Sea Basin^9–12^, the Baltic Sea Basin^13–15^, the Cariaco Basin^16,17^, and submarine caves, such as the Bahamian blue holes^18^, the Belize Blue Hole^19^, and the Sansha Yongle Blue Hole^20^.

In O_2_-deficient regions, microbial reactions control key steps in carbon, nitrogen, and sulfur transformation under successional redox gradients extending throughout the water column^21^. NO_3_^−^ is the most energetically favorable terminal electron acceptor for anaerobic respiration, prompting the development of a dynamic nitrogen cycle^4,5,22^. Much of the nitrogen loss in the ocean (30–50%) occurs in OMZs^23^. Heterotrophic denitrification and autotrophic anaerobic ammonium oxidation (anammox) are generally responsible for fixed nitrogen loss^24–31^. Dissimilatory NO_3_^−^ reduction to NH_4_^+^ (DNRA) takes place under suboxic or anoxic conditions and has the potential to moderate fixed nitrogen loss and to regenerate redox couples (NO_2_^−^ and NH_4_^+^) for anammox^5^. In OMZs, heterotrophic NO_3_^−^ reducers supply significant amounts of both NH_4_^+^ (via organic matter decomposition) and NO_2_^−^ (via NO_3_^−^ reduction) to the anammox process, suggesting a possible link between anammox and denitrification^29^. The relative contributions of denitrification and anammox to nitrogen loss from OMZs might depend on organic matter input, as well as on the availability of fixed nitrogen^29,30,32–34^. Sulfur cycling also plays an essential role in O_2_-deficient waters, coupling the production and consumption of H_2_S^4,6,7^. The γ-Proteobacterial clade SUP05 couple water column H_2_S oxidation to NO_3_^−^ reduction; these bacteria are widespread in the sulfidic waters at the bases of H_2_S/NO_3_^−^ transition zones in OMZs, including the Arabian Sea^35^, the ETSP^4,36,37^, the Peru Upwelling Region^6^, the Eastern Tropical North Pacific (ETNP)^38^, the Bay of Bengal^39^, the Cariaco Basin^40^, as well as the Baltic and Black Seas^41^. Chemolithoautotrophic sulfur-oxidizing and denitrifying ε-Proteobacteria, such as the *Sulfurimonas* subgroup, are most abundant under higher sulfidic water conditions, such as the Namibian Shelf^7^, the Cariaco Basin^40^, and the Baltic and Black Seas^41^. Denitrification and H_2_S oxidation might create an upper limit on the escape of H_2_S from anoxic waters, as well as provide autotrophic organic carbon resources, namely dark primary production. Thus, in O_2_-deficient waters, metabolically versatile microorganisms create complex networks of carbon-, nitrogen-, and sulfur-transforming reactions, which remain to be determined.

**Figure 1:**
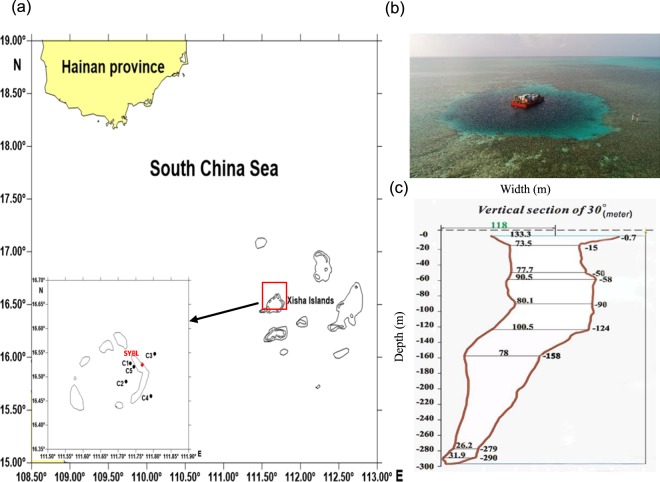
(**a**) Location of sampling sites in the Sansha Yongle Blue Hole and the surrounding regions. (**b**) Arial view of the Sansha Yongle Blue Hole. (**c**) Vertical cross-section of the Sansha Yongle Blue Hole.

The Sansha Yongle Blue Hole is located in the Yongle Atoll of the Xisha Islands, South China Sea. The cave entrance is shaped like a comma and has an average width of 130 m (Fig. 1)^42^. The physiochemical characteristics are presented in detail in our parallel hydrochemical study^20^. Briefly, the blue hole has a sharp chemocline and sulfidic bottom waters. The O_2_ concentrations at surface layer in the blue hole were nearly equivalent to the maximum O_2_ concentrations in the euphotic layer of the surrounding open sea, as well as to the maximum O_2_ concentrations in the surface layers of global OMZs^5–7,43^. The O_2_ concentration declined below the detection limit (<1 μmol l^−1^) at 100 m using the Winkler method. The primary NO_2_^−^ maximum (PNM, 0.4 µmol l^−1^) was identified at 30 m, and the secondary NO_2_^−^ maximum (SNM, 0.2 µmol l^−1^) was identified at 90 m. The NH_4_^+^ concentration began to increase at 90 m, increasing to ~100 µmol l^−1^ at 150 m. This concentration was maintained throughout the bottom layer waters. Similar to NH_4_^+^, H_2_S concentration increased noticeably from ~10 µmol l^−1^ at 100 m, up to ~48 µmol l^−1^ in the deeper, euxinic waters (≥150 m) that was probably due to the much reduced ventilation. In the blue hole, a suboxic zone was identified at ~90 m, where NO_3_^−^ (0.8 µmol l^−1^), NO_2_^−^ (0.2 µmol l^−1^), O_2_ (13.4 µmol l^−1^), H_2_S (0.03 µmol l^−1^), and NH_4_^+^ (3.9 µmol l^−1^) co-existed. O_2_-free condition and trace amounts of NO_3_^−^ and NO_2_^−^ were observed between 100 and 300 m. These properties notably differed from other OMZs, including the ETNP, the OMZ off Chilean in the South Pacific Ocean, and the Arabian Sea, which are typically O_2_-free but NO_2_^−^-rich^44^. The H_2_S and NH_4_^+^ within the blue hole anoxic zones were several times higher than levels observed below the oxycline in the Cariaco Basin^40^, the Baltic and Black Seas^41^, and the OMZ off Peru in the South Pacific Ocean^6^, where trace amounts of NO_3_^−^ were also detected. Particulate organic carbon (POC) concentration was low in the blue hole when compared to the Baltic sea, indicating that the blue hole had a poor nutrient input^45^. The blue hole is ~7 km and 70 km from Jinqing Island and Yongxing Island, respectively, and ~400 km south of Sanya^42^. Therefore, the anthropogenic activity has minimal influence. Together, the special geographical location and hydrochemical dynamics—in terms of the high levels of H_2_S and NH_4_^+^, as well as low levels of NO_3_^−^, NO_2_^−^ and POC—of the blue hole might allow for distinct microbial community with diverse metabolic function to be sustained.

In this study, 16S rRNA amplicons and metagenomic analyses were utilized to determine the microbial composition and vertical distribution patterns throughout the chemical gradient profiles in the Sansha Yongle Blue Hole and the open sea. We also characterized the genomic capacity of carbon, nitrogen, and sulfur pathways, as well as linkages to physical, chemical, and biological distribution patterns. This multidisciplinary investigation will inform a new framework to explore the responses and plasticity of marine ecosystems to O_2_ deficiency, which is expanding, intensifying, and occurring at shallower depths due to climate change^46,47^.

## Results and Discussion

### Microbial community structures

Multiple sites were sampled across a range of depths for direct comparison: one blue hole site (SYBL), two ocean sites (C3 and C4), and three lagoon sites (C1, C2, and C5; Fig. 1; Table 1). We successfully sequenced 40 PCR samples, and high-quality sequence reads were generated for further analysis. Overall, 16, 596 operational taxonomic units (OTUs) were identified across all samples, with some samples having up to 3, 595 OTUs.

**Table 1:** Details of the sampling sites in the Sansha Yongle Blue Hole and the surrounding regions.

Site	Location	Depth(m)	Sampling depth (m)	Description
SYBL	16.52°N, 111.77°E	300	0, 10, 30, 50, 70, 80, 90, 100, 125, 150, 200, 300	Sansha Yongle Blue Hole
C1	16.53°N, 111.73°E	17	0, 10	Lagoon
C2	16.49°N, 111.72°E	40	0, 10, 30	Lagoon
C5	16.52°N, 111.74°E	30	0, 10, 30	Lagoon
C3	16.55°N, 111.80°E	>600	0, 10, 30, 50, 75, 100, 150, 200, 300	Open sea
C4	16.46°N, 111.79°E	>600	0, 10, 30, 50, 75, 100, 150, 200, 300	Open sea

**Figure 2:**
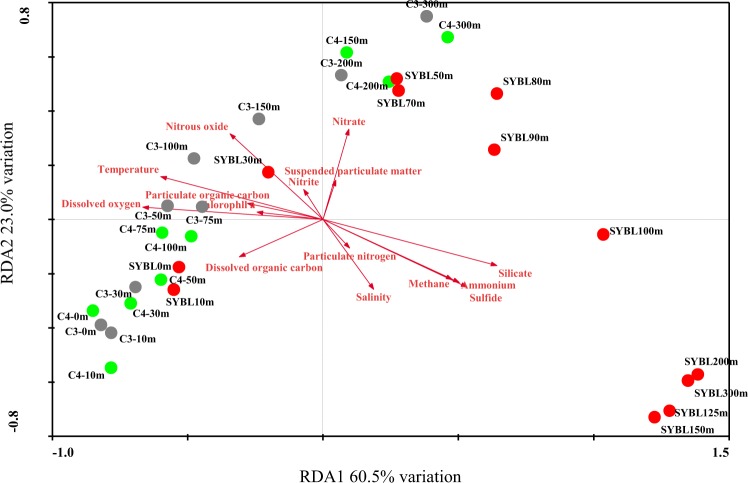
Redundancy analysis (RDA) of 70 microbial phyla from the Sansha Yongle Blue Hole and surrounding regions, based on 16S rRNA amplicon sequences.

**Figure 3:**
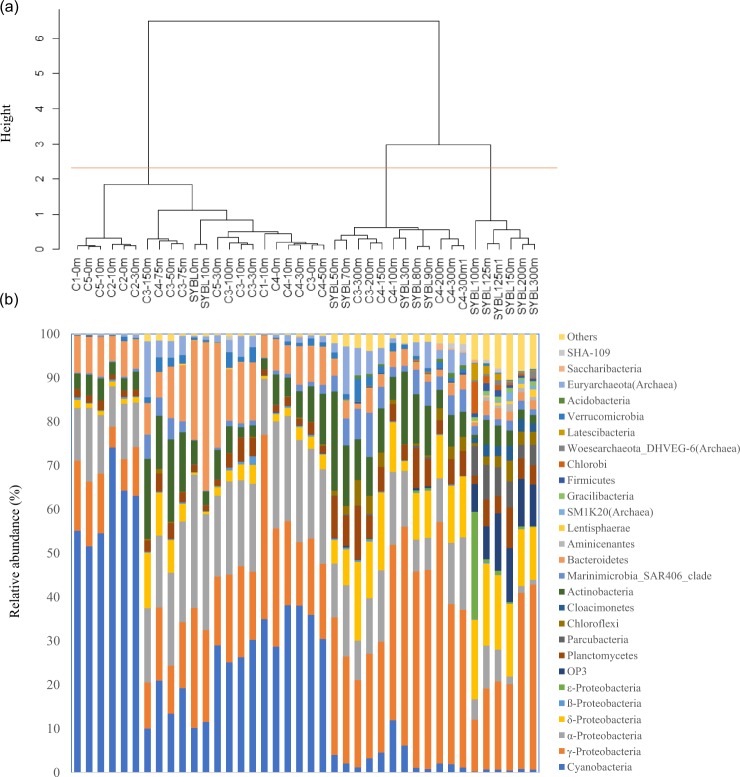
(**a**) Cluster analysis and (**b**) relative abundance of microbial phyla from the Sansha Yongle Blue Hole and surrounding regions, based on 16S rRNA amplicon sequences.

Based on redundancy analysis (RDA, Fig. 2), environmental data explained 83.5% of total community variation of the Sansha Yongle Blue Hole and surrounding open sea waters at the phylum level, where RDA1 explained 60.5% of the variation and RDA2 explained 23.0% of the variation. O_2_ was the most important factor affecting the microbial assemblages, and significantly explained 44.8% of the total variation, followed by silicate (44.2%), temperature (38.9%), H_2_S (34.8%), NH_4_^+^ (31.2%), CH_4_ (28.2%), nitrous oxide (N_2_O, 22.3%), NO_3_^−^ (12.8%), dissolved organic carbon (DOC, 12.4%), salinity(11.7%), POC (9.2%), and chlorophyll *a* (7.3%) (*P* < 0.05). On the basis of the cluster analysis, all samples fell into three groups at the phylum level (Fig. 3a). The first group consisted of samples located between 0 m and 30 m at C5, 0 m and 10 m at C1, 0 m and 30 m at C2, 0 m and 10 m at SYBL, 0 m and 150 m at C3, and 0 m and 75 m at C4. The samples at SYBL, C3, and C4 within this group had high levels of O_2_, DOC, POC, and chlorophyll *a* (Fig. 2). High levels of cyanobacteria were also observed within this depth range (Fig. 3b). The theoretical euphotic layer (1% of surface irradiance) was 51.2 m at SYBL and 80.8 m at C4, suggesting the samples in the first group at SYBL and C4 were located above the euphotic layer. Thus, these samples were characterized by high primary productivity and O_2_ enrichment via a light-driven process. The second group included samples located between 200 m and 300 m at C3, 100 m and 300 m at C4, and 30 m and 90 m at SYBL. These samples were from the depths with lower O_2_ concentration and higher NO_3_^−^ level, when compared with the first group, implying NO_3_^−^ accumulation and transformation. Samples in the third group were distributed among the anoxic bottom layer of the blue hole (100–300 m) and were characterized by high levels of H_2_S, NH_4_^+^, and CH_4_, suggesting highly reductive. The oxic-to-suboxic zone (30–90 m) in the blue hole displayed a similar microbial composition with deep waters of the surrounding open sea when compared with the anoxic bottom layer. This result is consistent with the observation that the functional capability of microbial communities at the shallow Landsort Deep of the Baltic Sea was similar to those of two deep communities: the 6 km-depth of a trench off Puerto Rico and the 1 km-depth of the Marmara Sea^47^. The similarities were both likely due to the stagnant conditions and hypoxia that shifted towards the surface of the water column^47^. Therefore, biochemical processes in deep waters might occur in shallow waters under O_2_ deficiency.

Samples were also recovered in three groups at the genus level, although some samples (C4-75 m, C3-50 m, and C3-150 m) were clustered with the deep-water open sea samples (Fig. S1). This suggested that the abundance and composition of the microbial genera shifted at the PNM (SYBL: 30 m; C3: 100–150 m; and C4: 75–100 m). Interestingly, the blue hole samples within each of the three branches formed separate sub-branches. This indicated that at high taxonomic level, microbial composition in the blue hole differed from that in the surrounding water. Microbial composition also varied throughout the water column, with distinct sub-divisions partitioned along the chemocline. Therefore, microorganisms occupied different niches in the blue hole that could be linked to different biogeochemical processes.

### Microbial composition and distribution

#### Surface layer

Based on the 16S rRNA amplicons, Cyanobacteria (10.9%), α-Proteobacteria (28.4%), γ-Proteobacteria (24.3%), Bacteroidetes (28.1%), and Actinobacteria (4.2%) were dominant at 0 m and 10 m in the blue hole (Fig. 3b). These populations are typical in marine environments, including the oxic surface waters overlying OMZs^46,48^. Consistently, metagenomic sequences of Cyanobacteria (25.4%), α-Proteobacteria (26.1%), γ-Proteobacteria (28.5%), and Bacteroidetes (5.4%) were dominant at 10 m in the blue hole (Fig. S2). The relative abundance of Cyanobacteria in the surface layer was 10.2% and 11.5%, which is similar to C3-150 m and C4-100 m. The extinction coefficient of visible light in the blue hole was higher than in the open sea and this rapid attenuation of light might limit cyanobacterial growth. We detected sequences affiliated with α-Proteobacterial class Rhodobacteraceae (relative abundance, 22.9% and 20.7%). Rhodobacteraceae-affiliated sequences were also abundant in the oxic surface waters overlying OMZs, including the Saanich Inlet and the ETSP^46^. Many Rhodobacteraceae species are known for their close associations with algal blooms, as well with particles^49–51^, and preferentially use labile organic substrates^51^. Bacteroidetes are the most abundant phylum in the world ocean after Proteobacteria and Cyanobacteria. In the blue hole, Flavobacteriales sequences accounted for a majority of Bacteroidetes and were most abundant at 0 m (22.2%) and 10 m (33.3%). This is consistent with the abundance of Bacteroidetes in other coastal areas (10–30%)^52^. Flavobacteriales are often associated with marine snow and marine phytoplankton blooms^50,53,54^. These bacteria attach to phytoplankton aggregates and efficiently degrade and preferentially consume high-molecular-mass organic matter as primary carbon and energy sources^51^.

#### Intermediate layer

Between 30 m and 90 m, the blue hole exhibited a sharp oxycline: from oxic (30–70 m), to hypoxic (80 m), and then to suboxic (90 m). The prevalent 16S rRNA amplicons across this transition included those affiliated with the γ-Proteobacteria (24.4–49.9%), Actinobacteria (11.3–22.6%), α-Proteobacteria (7.3–16.3%), Planctomycetes (3.5–9.6%), Euryarchaeota (0.2–10.9%), SAR406 (1.3–6.1%), and Cyanobacteria (0.8–6.2%) (Fig. 3b). Metagenomic sequences of Cyanobacteria (8.1%, 1.2%), α-Proteobacteria (28.8%, 9.3%), γ-Proteobacteria (47.9%, 40.3%), Euryarchaeota (0.7%, 1.5%), and Actinobacteria (0.9%, 2.5%) were also dominant at 30 m and 90 m in the blue hole, respectively (Fig. S2). In the blue hole, γ-Proteobacterial genus *Alteromonas* 16S rRNA sequences were abundant throughout the water column, especially at 30 m, 80 m, and 90 m (23.3–34.9%). *Alteromonas* species are widespread in shallow and deep waters of global oceans, including the ETNP OMZ^55–57^. *Alteromonas* species are particle-associated microaerophilic bacteria. In addition to relying on phytoplankton-derived organic matter for survival, *Alteromonas* species can also use NO_3_^−^ as a nitrogen source^58^. SAR406 might participate in sulfur cycling via dissimilatory polysulfide reduction or sulfide oxidation^59^. The abundance of 16S rRNA sequences affiliated with SAR406 was 5.4–6.1% at 70–90 m in the blue hole, equivalent to SAR406 abundances at 150 m and 300 m at C3 and C4 (4.9–10.1%). SAR406 sequences were also highly abundant in the global OMZs^46,59^. In addition, 16S rRNA sequences affiliated with the methane-oxidizing archaean Marine Group II (phylum Euryarchaeota, class Thermoplasmata) were highly abundant in the blue hole at 70 m and 90 m (10.9% and 6.0%, respectively). These levels were comparable to Marine Group II abundance at 150–300 m at C3 (5.2–12.7%). The nitrite-oxidizing autotrophic *Nitrospina* (phylum Nitrospirae) was abundant between 50 m and 90 m in the blue hole (3.3–7.2%). The greatest *Nitrospina* abundance was at 90 m in the blue hole (7.2%), at 300 m at C3 (8.6%), and at 300 m at C4 (4.2%), suggesting that this genus occupied a wide range of niches.

#### Anoxic bottom layer

In the anoxic deeper waters of the blue hole (100–300 m), O_2_ was <1.0 µmol l^−1^, concentrations of H_2_S, NH_4_^+^, SiO_3_^2−^, PO_4_^3−^, and CH_4_ increased with depth, and only trace amounts of NO_2_^−^ and NO_3_^−^ were detected^20^. The microbial composition in this water layer was distinct, with the most abundant 16S rRNA amplicon sequences affiliated with the γ-Proteobacteria (11.9–42.2%), δ-Proteobacteria (12.0–18.7%), *Candidatus* OP3 (6.3–13.1%), Planctomycetes (2.0–9.3%), and *Candidatus* Parcubacteria (3.0–7.8%) (Fig. 3b). Also, metagenomic sequences of γ-Proteobacteria (67.4%) were dominant at the bottom waters of the blue hole (Fig. S2). The 16S rRNA amplicon sequences associated with the δ-Proteobacteria primarily included SO_4_^2−^ reducers, such as species from Desulfarculaceae, Desulfobulbaceae, and Desulphobacteraceae. We also identified 16S rRNA amplicon sequences affiliated with heterotrophic γ-Proteobacterial *Pseudoalteromonas* (29.6% at 200 m, 21.9% at 300 m) and *Alteromonas* (15.3% at 300 m, ~11.0% at 125–150 m), ε-Proteobacterial sulfur oxidizer *Arcobacter* (24.1% at 100 m), and phototrophic *Prosthecochloris* (Chlorobi, 7.2% at 100 m).

The O_2_-deficient environments often display ecologically specialized microbial populations, potentially mediating organic carbon turnover and syntrophic interactions. In the bottom layer waters of the blue hole, the clades of syntrophic taxa identified could potentially degrade lignocellulosic plant material or algal-derived complex organic polymers in order to produce hydrogen (H_2_), including *Fibrobacter succinogenes* (phylum Fibrobacteres)^60^, Latescibacteria^61^, and Firmicutes. The syntrophic bacteria also included taxa that convert small molecular compounds, such as glucose, pyruvic acid, short chain fatty acids, and glycerol to acetate and H_2_ for CH_4_ production—e.g., Thermotogae, Spirochaetae, *Sebaldella termitidis* (Fusobacteria), *Elusimicrobium minutum* (Elusimicrobia), Cloacimonetes, Atribacteria, *Candidatus* Acetothermus autotrophicum (Acetothermia), and *Candidatus* Hydrogenedentes^62^. Therefore, the blue hole represented a great amount phylogenetic and functional diversity of microbial communities that could drive matter and energy transformation throughout the water column.

### Nitrogen-based metabolic potential

#### NH_4_^+^ production

**Figure 4:**
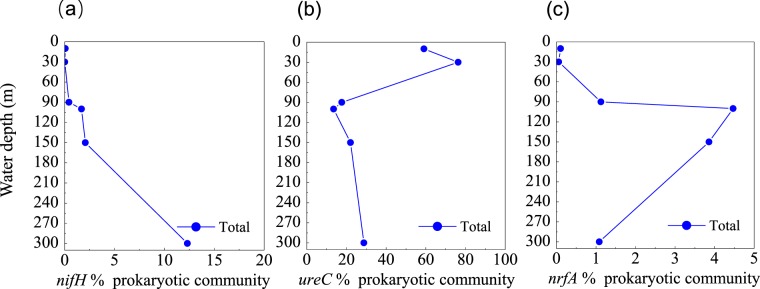
Profile of the abundances of (**a**) *nifH*, (**b**) *ureC*, and (**c**) *nrfA* genes for NH_4_^+^ production, from the Sansha Yongle Blue Hole. The abundance of functional genes was shown relative to the putative single copy per organism of RNA polymerase subunit B (*rpoB*). Abundances per gene are normalized to gene length.

NH_4_^+^ is a central component of the marine nitrogen cycle. Sources of marine NH_4_^+^ include the degradation of organic nitrogen compounds, ammonification, N_2_ fixation, hydrolysis of urea, and DNRA^63^. We identified genes encoding molybdenum-iron nitrogenase (MoFe, *nifHDK*) in the blue hole, affiliated with Cyanobacteria, Chlorobi, Bacteroidetes, Proteobacteria, Firmicutes, and Verrucomicrobia. The gene of *nifH* increased with depth, indicating that the microbial fixation of N_2_ was more common in deep waters of the blue hole (Fig. 4a). We also identified *ureABC* genes, which encode urease, associated with Thermoplasmata, Thaumarchaeota, Cyanobacteria, Actinobacteria, and Proteobacteria. High abundance of *ureC* gene at 10 m and 30 m (59.1% and 76.3%) was associated with the clades of Cyanobacteria and *Alteromonas australica* (γ-Proteobacteria) (Fig. 4b). The gene of *nrfA*, encoding dissimilatory ammonia-forming nitrite reductase, peaked at 100 m (4.5%), and was primarily detected in γ-Proteobacteria and δ-Proteobacteria (Fig. 4c).

#### Denitrification

**Figure 5:**
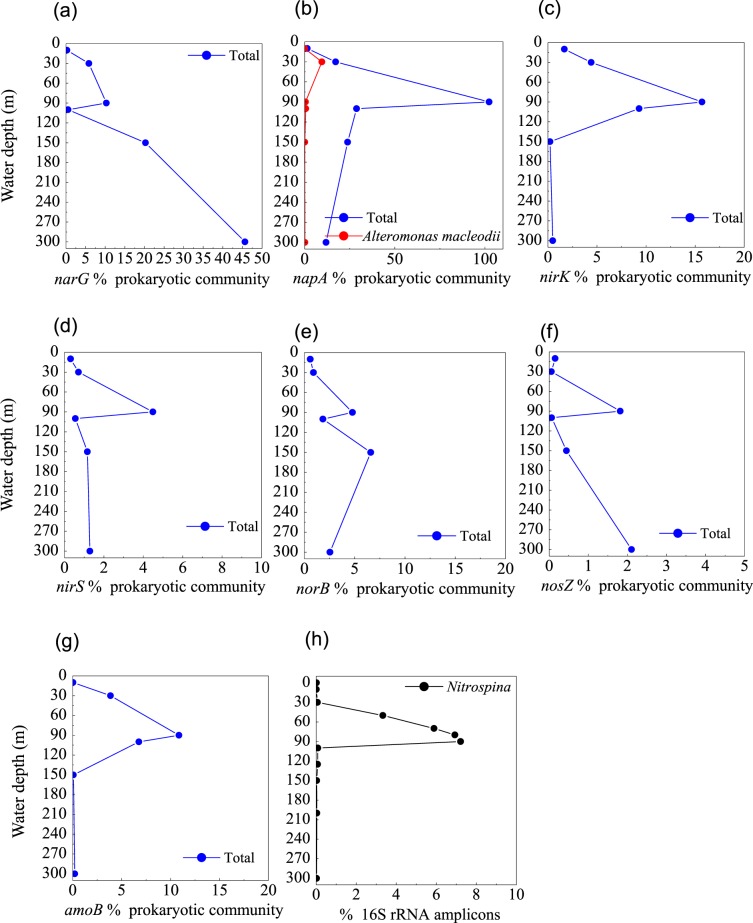
Profile of the abundances of (**a**) *narG*, (**b**) *napA*, and (**c**) *nirK*, (**d**) *nirS*, (**e**) *norB*, and (**f**) *nosZ* genes for denitrification, (**g**) *amoB* gene for nitrification, (**h**) the relative abundance of *Alteromonas* based on 16S rRNA amplicon sequences from the Sansha Yongle Blue Hole.

**Figure 6:**
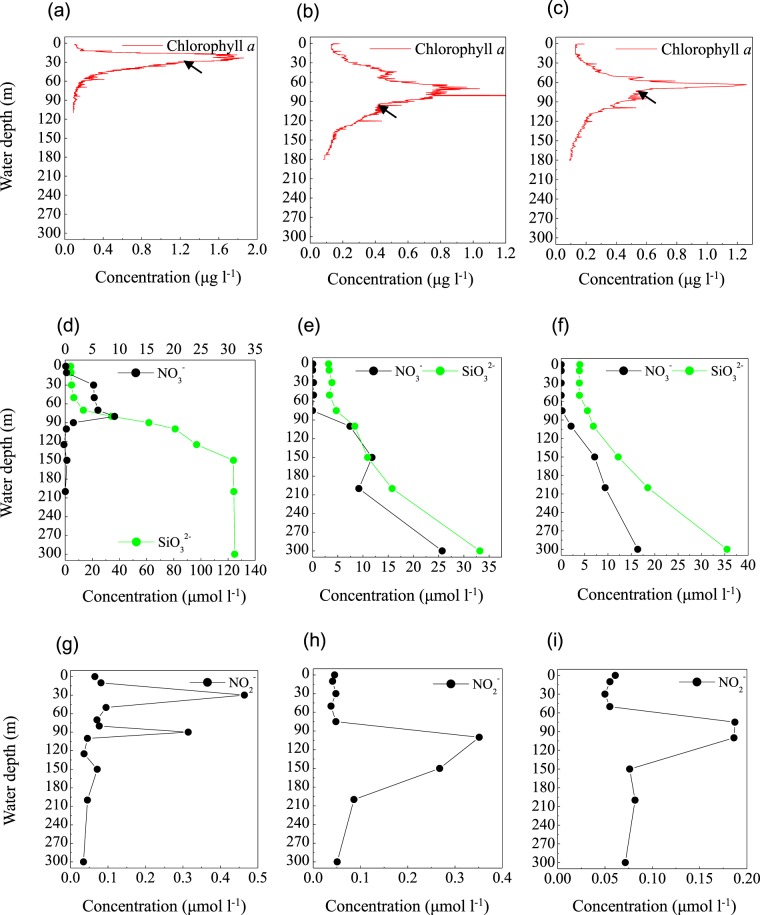
Chlorophyll *a* concentration, NO_3_^−^ and SiO_3_^2−^ concentrations; NO_2_^−^ concentration from the Sansha Yongle Blue Hole (**a**,**d**,**g**); C3 (**b**,**e**,**h**); C4 (**c**,**f**,**i**). The arrows indicate the onset of PNM. (**d–i**) were based on our parallel hydrochemical study^20^.

The first step in denitrification—NO_3_^−^ reduction to NO_2_^−^—can be catalyzed by nitrate reductases. The metagenomes in the blue hole were enriched in the *narG* gene, which encodes respiratory nitrate reductase, and the *napA* gene, which encodes periplasmic nitrate reductase at 90 m, accounting for 10.3% and 102.3% of prokaryotic community, respectively (Fig. 5a,b). This corresponded well with a reduction in NO_3_^−^ concentration and the SNM at 90 m (Fig. 6d,g), implying NO_3_^−^ reduction activity. More than 100% of the prokaryotic community contained the *napA* gene, implying multiple copies per genome in some members. At 90 m, the *narG* sequences primarily matched α-Proteobacteria, as well as γ-Proteobacteria (Enterobacteriaceae and a thioautotrophic gill symbiont of *Bathymodiolus septemdierum*). The proportion of *narG* gene was much higher at 150 m and 300 m than at 90 m, Alteromonadales and unclassified bacteria contributed to the high abundance, however, the capacity for these populations to perform NO_3_^−^ reduction under trace NO_3_^−^ and NO_2_^−^ conditions is unknown. The *napA* gene sequences primarily matched γ-Proteobacteria (*Aeromonas hydrophila*, *Thiolapillus*, endosymbionts from an unidentified scaly snail isolate Monju, and *Candidatus* Thioglobus sp. EF1), ß-Proteobacteria (*Sulfuricella denitrificans* and *Burkholderia xenovorans*), and ε-Proteobacterial genus *Arcobacter*. In addition, *napA* gene from *Alteromonas macleodii* accounted for up to half of all *napA* gene sequences at 30 m, suggesting that these species might be responsible for the PMN formation (Fig. 5b).

Genes that could accomplish other steps of denitrification were identified from a consortium of diverse members, indicating a high genomic potential for complete denitrification to N_2_ in the blue hole. These denitrification genes encoded copper-containing nitrite reductase (*nirK*), iron-containing nitrite reductase (*nirS*), nitric oxide reductase (*norB*), and nitrous oxide reductase (*nosZ*), but were not detected at high frequencies in comparison to *narG* and *napA*. NO_2_^−^ reduction to NO is mediated by *nirK/nirS*, and the greatest number of *nirK* gene was detected at 90 m, where it was present in ∼16% of the prokaryotic community (Fig. 5c). Marine Group I Thaumarchaeota was the dominant *nirK*-containing population. The *nirS* gene was present in a lower percentage of the community than *nirK*, but were also most abundant at 90 m (4.5%) (Fig. 5d). NO reduction to N_2_O is mediated by *norB,* which was affiliated with γ- and ε-Proteobacteria in this study, achieving two maxima at 90 m and 150 m, respectively (Fig. 5e). N_2_ production from N_2_O is mediated by *nosZ* gene, which peaked at 90 m and 300 m in abundance (Fig. 5f). The Rhodospirillaceae-related *nosZ* was abundant at 90 m, corresponding well to the upper *nosZ* maximum, and Flavobacteriaceae-related *nosZ* was abundant at 300 m. Flavobacterial *nosZ* was also detected in marine O_2_-deficient zones in the ETNP^64^. N_2_O produced in shallow water is likely to be released into the atmosphere^65^. However, the N_2_O concentration from the surface layer of the blue hole was similar to open sea surface water^20^, suggesting that a new balance may have been established.

#### Nitrification

Ammonia monooxygenase catalyzes NO_2_^−^ production via NH_4_^+^ oxidation. The *amoB* gene encoding ammonia monooxygenase was primarily associated with *Nitrosopumilus* (Thaumarchaeota), reaching a maximum of 10.9% of the prokaryotic community at 90 m in the blue hole (Fig. 5g). This maximum in *amoB* corresponded well with the SNM and the onset of the NH_4_^+^ increase, implying that NO_2_^−^ accumulation occurs via NH_4_^+^ oxidation. The relative abundance of *Nitrospina* based on 16S rRNA amplicons in the blue hole, increased with depth (0–90 m) and in parallel with NO_3_^−^ concentration, indicating that the NO_2_^−^-oxidizing chemoautotroph might produce the observed NO_3_^−^ (Figs. 5h and 6d). *Nitrospina* was also the main driver of NO_2_^−^ oxidation in the upwelling areas of the Eastern South Pacific, increasing in abundance with depth^66,67^. However, the *nxr* gene (encoding a nitrite-oxidizing enzyme, nitrite oxidoreductase) could not be detected in the metagenomes from the blue hole water column, suggesting a low abundance of *Nitrospina*. The relative abundance of *Nitrospina* might be overestimated based on measured 16S rRNA amplicons.

#### Anammox

To date, *Scalindua* is the only genus of anammox bacteria found in marine environments^68^. Low abundance of 16S rRNA amplicons matching *Scalindua* was present at the water depth between 80 m and 100 m (0.01–0.02%) in the blue hole, where NH_4_^+^ and NO_2_^−^ overlapped at 80 m and 90 m, and NO_2_^−^ began to disappear at 100 m. However, *Scalindua*-related sequences were not recovered in the metagenomes from the blue hole. This suggested that denitrification could be the dominant pathway of N_2_ formation in the blue hole, considerably outpacing anammox. The NO_2_^−^ depletion in the bottom waters could limit the anammox pathways, although high NH_4_^+^ concentration was detected. Moreover, H_2_S could also inhibit the anammox activity^69^. This phenomenon was also detected in the OMZ off Peru in association with a giant H_2_S plume^6^. In contrast, abundant anammox activity was detected in the suboxic zone of the Black Sea where high levels of NO_3_^−^ and NO_2_^−^ were present^69^.

#### The primary NO_2_^−^ maximum (PNM)

**Figure 7:**
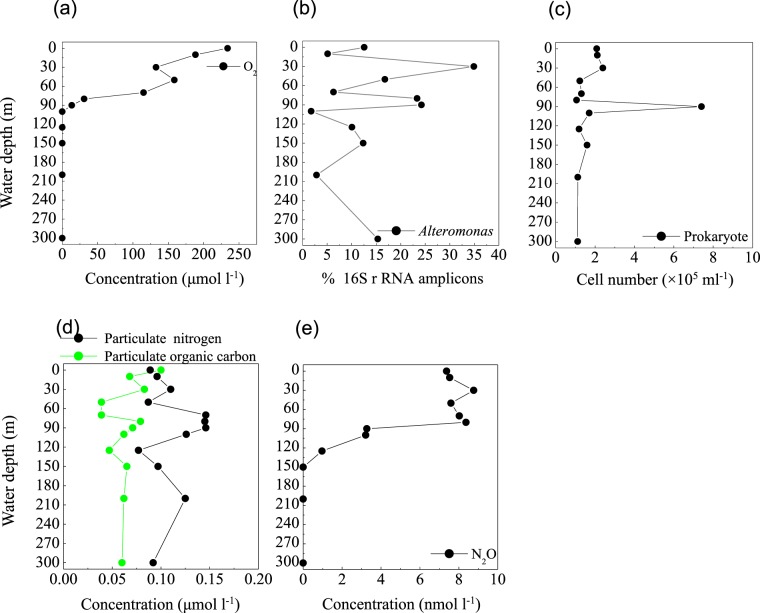
Profile of (**a**) O_2_ concentration, (**b**) the relative abundance of *Alteromonas* based on 16S rRNA amplicon sequences, (**c**) prokaryotic cell number, (**d**) Particulate nitrogen and particulate organic carbon concentrations, and (**e**) N_2_O concentration from the Sansha Yongle Blue Hole. (**a**,**d**,**e**) were based on our parallel hydrochemical study^20^.

In the blue hole, based on the depth of the chlorophyll *a* peak base (~50 m) (Fig. 6a) and the onset of SiO_3_^2−^ accumulation (50 m) (Fig. 6d), we first hypothesized that the PNM would be located at ~50 m, consistent with the theoretical euphotic limit (51.2 m), and similar positions observed for the PNM in C3 (Fig. 6b,e,h) and C4 (Fig. 6c,f,i). Unexpectedly, the primary maxima of both NO_2_^−^ and N_2_O were identified at 30 m, close to the depth of the chlorophyll *a* peak (Figs. 6g and 7e). At 30 m, we also identified peaks for a primary O_2_ minimum (~130 μmol l^−1^, Fig. 7a), a primary POC, and particulate nitrogen (PN) (Fig. 7d). However, the NO_3_^−^ concentration peaked at the bottom of the PNM (80 m, Fig. 6d). These are all classic signals for denitrification. Indeed, *Alteromonas* species were maximally abundant (34.9%) at 30 m in the blue hole (Fig. 7b). Of these species, one particularly abundant species (32.1%) with a 99% identity to *Alteromonas macleodii* was identified (Fig. S3a). Moreover, the *NapA* gene from *Alteromonas macleodii* accounted for up to half of all *napA* gene sequences at 30 m (Fig. 5b). Therefore, we speculated that, at 30 m in the blue hole, photoautotrophs formed large amounts of POC, which fueled microbial growth and aerobic respiration, leading to O_2_ deficency. In addition, phytoplankton particles generate microscale oxyclines for suboxic or anoxic respiration in oxygenated waters^57^. Based on the formula of Stief *et al*.^70^, given an ambient O_2_ of 130 µmol l^−1^ at 14 °C, O_2_ concentration at the center of the diatom aggregate was ~40 µmol l^−1^, comparable to the value of 39 µmol l^−1^ that inhibits NO_3_^−^ reduction^71^. Reasoning that the O_2_ solubility decreases with increasing temperature, at higher temperature of 25.6 °C at 30 m^20^, O_2_ concentration would be even lower within the organic aggregates. At such low O_2_ concentration, *Alteromonas* species might reduce NO_3_^−^, leading to the accumulation of NO_2_^−^ in oxygenated waters. Experimental conditions have measured NO_3_^−^ reduction at low O_2_ concentrations, which presumably matches to anoxic micro-environments^71,72^. Isolating and culturing an *Alteromonas macleodii* strain from 30 m in the blue hole revealed that this strain grew statically in diluted liquid 2216E marine medium (0.5 g yeast, 2.5 g tryptone, 1-L sea water) supplemented with 300 µmol l^−1^ NaNO_3_ for 3 d, and NO_2_^−^ accumulation was evident (5.6 µmol l^−1^, unpublished data). This suggested that *Alteromonas macleodii* could perform NO_3_^−^ reduction in the stagnant water. Altogether, in the O_2_-limited blue hole, a PNM at shallow water depth was identified and the denitrification activity of *Alteromonas* species might play important role in generating the PNM. Additionally, denitrification by aggregate-associated bacteria may shift the PNM towards the chlorophyll *a* peak in an O_2_-deficient marine system, which may previously have been overlooked. In addition, a primary NH_4_^+^ maximum was detected between 20 m and 80 m in the blue hole^20^. Low abundance of *amoB* gene sequences coupled with NH_4_^+^ substrate at 30 m could also partly contribute to NO_2_^−^ accumulation (Fig. 5g).

#### The secondary NO_2_^−^ maximum (SNM)

In low O_2_ environments, a SNM is often detected below the PNM^73^. NO_2_^−^ in the SNM is mainly produced by dissimilatory NO_3_^−^ reduction, an alternative respiratory mechanism that becomes favorable when O_2_ is limited. We observed a SNM in the blue hole at 90 m in close proximity to the base of the NO_3_^−^ maximum (where O_2_ concentration had decreased to 13.4 µmol l^−1^) (Figs. 6g and 7a). The maximal POC and PN concentrations occurred in this layer, as well as highest abundance of prokaryotes (Fig. 7d,c). Both *narG* and *napA* genes present in heterotrophic Proteobacteria were also enriched at 90 m in the blue hole (Fig. 5a,b). Thus, at 90 m in the blue hole, O_2_-deficient condition and a high particle load might lead to an alternative respiration prevalent, with NO_3_^−^ as an electron acceptor. In addition, at 90 m, populations containing NO_3_^−^ reducing genes also harbored sulfur-oxidizing genes, including γ- Proteobacteria (thioautotrophic gill symbiont of *Bathymodiolus septemdierum*, and *Candidatus* Thioglobus), ε-Proteobacteria (*Arcobacter* and *Sulfurimonas*), and Chlorobiaceae. Therefore, the sulfur-driven chemolithotrophic denitrification could also be a crucial method for SNM formation. In addition, *amoB* gene reached a maximum of 10.9% of the community at 90 m, which might also be partly responsible for the NO_2_^−^ accumulation (Fig. 5g).

### Sulfur-based metabolic potential

#### Sulfate reduction

**Figure 8:**
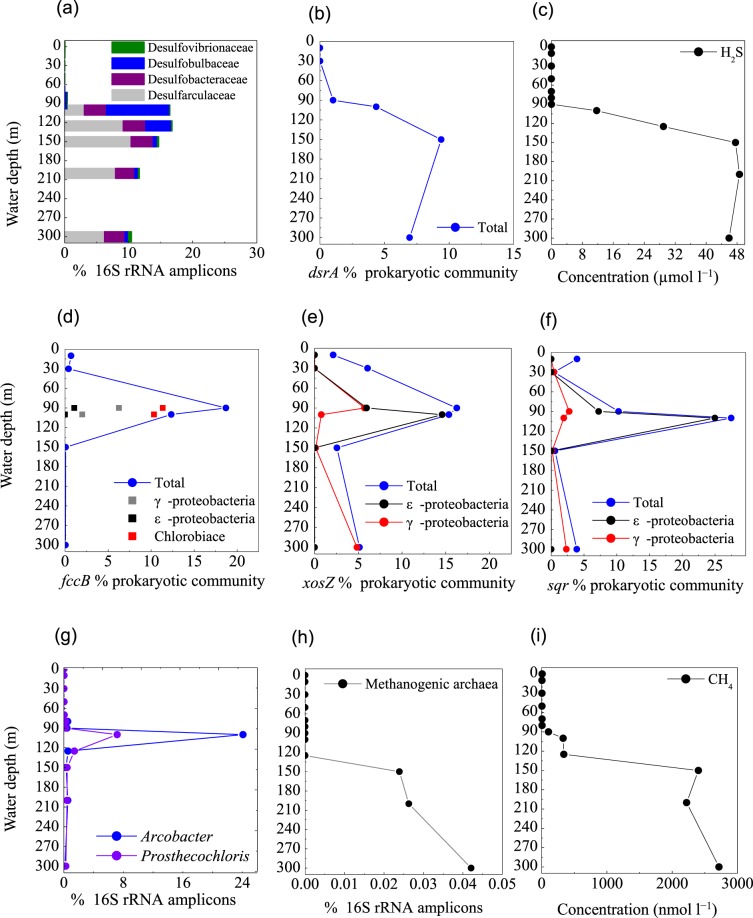
Profile of the abundances of (**a**) representative SO_4_^2−^-reducing populations, (**b**) *dsrA* gene for sulfate reduction, (**c**) H_2_S concentration, (**d**) *fccB*, (**e**) *soxZ*, and (**f**) *sqr* genes for sulfur oxidization, (**g**) representative sulfur-oxidizing denitrifiers, (**h**) Methanogenic archaea, (**i**) CH_4_ concentration, from the Sansha Yongle Blue Hole. (**c**,**i**) were based on our parallel hydrochemical study^20^.

Under O_2_ depletion, both episodic plumes of H_2_S in continental shelf regions and permanent H_2_S under sulfidic conditions are produced by SO_4_^2–^-reducing bacteria from SO_4_^2–^ ^4,6,7^. Based on 16S rRNA amplicons, diverse SO_4_^2−^-reducing populations were detected at 90 m, accounting for 0.4% of total prokaryotes in the blue hole, which increased rapidly between 100 m and 300 m (10.6–16.7%). These SO_4_^2−^reducers included *Desulfatiglans* (family Desulfarculaceae, 3.0–10.2%) and *Desulfurivibrio* (family Desulfobulbaceae, 0.1–2.6%). In addition, an unclassified genus in the Desulfobacteraceae (2.4–3.9%) and an unclassified genus in the Desulfobulbaceae (0.4–5.8%) were also identified. Among these taxa, *Desulfococcus* (0.04–0.21%) and *Desulfovibrio* (0.05–0.21%) were also detected in OMZ waters off the Chilean Coast^4^. The relative abundances of sequences associated with the Desulfovibrionaceae, Desulfarculaceae, Desulfobulbaceae, and Desulphobacteraceae were represented in Fig. 8a. In good agreement with this data, metagenomic results suggested that gene sequences encoding dissimilatory sulfite reductase (*dsrA*) were present in high proportions between 90 m and 300 m (1.0–9.3% of the community) (Fig. 8b). In contrast, SO_4_^2−^-reducing population represented only ~0.04% between 0 m and 80 m in the blue hole, and 0.1–0.2% in the surrounding regions. The *dsrA* distribution was paralleled by SO_4_^2−^-reducing populations and the H_2_S concentration (Fig. 7c) in the blue hole. Therefore, SO_4_^2−^ reduction in the water column is an important pathway, and might contribute to large volumes of H_2_S, creating a sulfidic zone as thick as ~200 m.

#### Sulfur oxidization

Clades of sulfur-oxidizing bacteria are particularly enriched at the oxic–anoxic interfaces, where O_2_, NO_3_^−^, and metal oxides are available as electron acceptors^6,7,19,74–76^. At these interfaces, H_2_S can be oxidized using the sulfide: quinone oxidoreductase enzyme (Sqr) and flavocytochrome c/sulfide dehydrogenases (Fcc), forming SO_3_^−^. The SO_3_^−^ can be further oxidized to SO_4_^2−^ by the adenylylsulfate reductase (Apr) and sulfate adenylyltransferase (Sat). Elemental sulfur and S_2_O_3_^2−^ are presumably oxidized to SO_4_^2−^ via the sulfur-oxidizing multienzyme complexes (Sox)^77^.

Mining the metagenomic data, we identified genes that could allow for dissimilatory sulfur oxidation, including genes that encode sulfide: quinone-oxidoreductase (*sqr*), flavocytochrome c/sulfide dehydrogenase (*fccABC*), sulfate adenylyltransferase (*sat*), adenylylsulfate reductase (*apr*) and sulfur-oxidizing multienzyme complexes (*soxABCD* and *soxYZ*) in the blue hole. These genes were detected in various combinations across diverse sulfur-utilizing taxa, primarily affiliated with γ-, ε-Proteobacteria, and Chlorobi. The greatest abundances of *fccB* and *soxZ* genes were present at the suboxic layer (90 m) (Fig. 8d,e), while *sqr* gene was present at the upper anoxic layer (100 m) (Fig. 8f), coinciding with a steep decline in H_2_S concentration^20^. This suggested that H_2_S oxidation may occur at the NO_3_^−^/NO_2_^−^-H_2_S transition in the blue hole. Further, *fccB* gene affiliated with Chlorobiaceae was comparable at 90 m and 100 m (~10% of community) (Fig. 8d). Genes encoding *fccB*, *sqr*, and *soxZ* affiliated with *Candidatus* Thioglobus (γ-Proteobacteria SUP05) were dominant at 90 m, while genes of *sqr* and *soxZ* affiliated with ε-Proteobacterial genera *Sulfurimonas* and *Arcobacter* were enriched at 100 m (Fig. 8d–f). Depth-specific patterns among different sulfur-metabolizing taxa might reflect differences in O_2_ sensitivity, as well as adaptations to varying energy substrates.

Chlorobiaceae species are phototrophic bacteria^78^. The high abundance of *fccB* gene(11.4% at 90 m and 10.3% at 100 m) and 16S rRNA amplicons (7.2% at 100 m), within the narrow layer might indicate that Chlorobiaceae members could potentially couple H_2_S oxidation to phototrophy, even at extremely low-light intensities (Fig. 8d,g). The Chlorobiaceae taxa contained sequences encoding nitrate reductase and nitrous reductase, as well as a RuBisCO-like protein for CO_2_ fixation. This suggested Chlorobiaceae could use NO_3_^−^ as a potential terminal electron acceptor for H_2_S oxidation, linked to CO_2_ assimilation via Calvin cycle for dark primary production.

The metagenomic data suggested that sulfur oxidizing genes found in *Candidatus* Thioglobus (SUP05) were enriched in the suboxic and anoxic zones of the blue hole. Genes of *sqr* (0.7% at 90 m, 0.2% at 100 m), *fccB* (6% at 90 m, 1.4% at 100 m), *soxZ* (5.0% at 90 m) and *napA* (0.03% at 90 m, 0.01% at 100 m) were recovered, implying that *Candidatus* Thioglobus may prefer to reside within suboxic zone. These results support finding from recent surveys indicate that γ-Proteobacterial SUP05 can oxidize sulfur by denitrification, and is most abundant in slight to moderate redoxclines, thereby linking sulfur cycling to N-loss pathways^4,12,37,38^.

In contrast to SUP05-related sequences, ε-Proteobacteria preferentially colonized anoxic and highly sulfidic environments in the blue hole. The *soxZ* gene related to *Sulfurimonas* occupied 13.8% of the community at 100 m, although a minor component of the 16S rRNA amplicons (0.4%) affiliated with *Sulfurimonas* was detected. In addition, up to half of the *norB* sequences were related to *Sulfurimonas* species, further suggesting that the sulfur-oxidizing genus *Sulfurimonas* also supported reductive nitrogen metabolism. *Sulfurimonas* species are also widespread in the sulfidic anoxic waters of the Benguela system off Namibia^7^, as well as in the anoxic waters of the Baltic Sea, the Black Sea^41^, and the Cariaco Basin^42^. The *sqr* sequences were present in high abundance in *Arcobacter* (7.2% at 90 m and 25.0% at 100 m). Correspondingly, 16S rRNA amplicons affiliated with *Arcobacter* were also most abundant at 100 m (24.1%) (Fig. 8g). Based on the alignment of these 16S rRNA sequences with previously published sequences in GenBank, one *Arcobacter*-affiliated sequence from the blue hole (23.9%) were 99% identical to gill epibionts of hydrothermal vent gastropods, and *Arcobacter* clones from the Saanich Inlet, from the near-shore anoxic basin, and from the costal oxycline, respectively. Another *Arcobacter*-affiliated sequence also had 96% identity to *Arcobacter nitrofigilis* and 95% identity to *Arcobacter sulfidicus* (Fig. S3b). *Arcobacter*-associated sequences were also found in the OMZs off Peru^6^ and the sulfidic Benguela system off Namibia^7^, accounting for ~2–10% in abundance. These species were identified as key organisms in the chemolithotrophic oxidation of H_2_S with NO_3_^−^^7^. The metagenomic data from our study indicated that *Arcobacter* species might perform denitrification (*nap*A, *nir*, *nor*), as well as oxidizing HS^–^/S^2–^ to S^0^ (*sqr*, *fccB*) and SO_3_^2−^ to SO_4_^2−^ (*soxACD* and *soxYZ*) for energy generation. Additionally, *Arcobacter-*affiliated sequences contained genes encoding clades of proteases, peptidases, and oligopeptidases, as well as enzymes critical for the oxidative tricarboxylic acid (TCA) cycle (citrate synthase). However, no glycerases were identified. This indicated that *Arcobacter* species used proteins, amino acids, propionates, and TCA cycle intermediates, but not carbohydrates^79^. We also identified gene sequences encoding key enzymes of the rTCA cycle for chemoautotrophic CO_2_ fixation, including citrate lyase (*aclB*), pyruvate flavodoxin oxidoreductase (*porA*), and 2-oxoglutarate-acceptor oxidoreductase (*oorA*). Therefore, *Arcobacter* species had the genomic capacity to grow chemolithoautotrophically via H_2_S or S_2_O_3_^2−^ oxidation that is linked to diverse steps of denitrification, as well as heterotrophically on various organic compounds. The metabolic versatility of *Arcobacter* might provide a competitive advantage in the energy-limited blue hole.

The microbial reduction of NO_x_ coupling to sulfur oxidation pathways has been documented in diverse taxa from the H_2_S/NO_3_^−^ transition zones in OMZs^4,6,35–41^. In the blue hole, sulfur-oxidizing denitrifiers—such as γ-, ε- Proteobacteria, and Chlorobiaceae—were enriched at 90 m and 100 m, supporting sulfur oxidation that is coupled to reductive nitrogen metabolism. It is obvious that sulfur-based denitrification occurs in this zone (90 m), where NO_3_^−^/NO_2_^−^ and H_2_S overlapped. Meanwhile, *amoB* gene from *Nitrosopumilus* (Thaumarchaeota) was recovered at 100 m, indicating that NH_4_^+^ oxidation could provide the NO_2_^−^ substrate necessary for denitrification, although this process is transient and cryptic, as trace NO_3_^−^/NO_2_^−^ was detected at 100 m. This is in good agreement with a previous report on the anoxic water at Landsort Deep of the Baltic Sea^47^. We speculate that H_2_S produced by heterotrophic sulfur reducers could support sulfur-driven chemolithotrophic denitrification, which mediates both nitrogen loss and H_2_S removal from the blue hole.

### CH_4_ cycle

In the blue hole, sequences associated with methanogens (phylum Euryarchaeota, order Methanomicrobiales and Methanosarcinales) were identified at 150–300 m, with a total abundance of 0.02–0.04% of total 16S rRNA amplicons (Fig. 8h). The total abundance of these taxa at 150–300 m was linear positively correlated with the concentration of CH_4_ (~2.4–2.7 µmol l^−1^, Fig. 8i, *r*  =  0.838). Gene encoding methyl-coenzyme M reductase (*mcrA*), the best diagnostic enzyme for anaerobic methanogenesis, was not found in the metagenomic data. This could be explained by low levels of archaeal 16S rRNA amplicons. However, metagenomic and metatranscriptomic data in the 300 m surface sediment revealed a *mcrA* gene belonging to Methanosarcinales, suggesting active methanogenesis (unpublished data). Based on this study’s 16S rRNA amplicons and metagenomic sequences, coupled with recent published literatures, we propose three methanogenic pathways in the bottom waters of the blue hole. (1) *Methanococcoides* and *Methermicoccus* adopt methylotrophic pathways, including one-carbon compound pathways such as methanol conversion to CH_4_^80^. Consistently, gene sequences for key enzymes were found, such as trimethylamine-corrinoid protein Co-methyltransferase and Methylated-thiol-coenzyme M methyltransferase. (2) *Methanosaeta* and *Methanosarcina* catalyze the acetoclastic pathway (acetate conversion to CH_4_)^81,82^. (3) The family Methanomicrobiaceae catalyzes the hydrogenotrophic pathway (H_2_ + CO_2_ → CH_4_)^83^. In terms of abundance, methylotrophic methanogenesis was the major pathway in the blue hole, in agreement with previous reports that some methanogens can survive in the presence of SO_4_^2-^ reducers by consuming noncompetitive methylated substrates^84^. In contrast, SO_4_^2−^ reduction processes could compete for these substrates, (e.g., H_2_ and acetate), potentially leading to a low proportion of sequences related to hydrogenotrophic and acetoclastic methanogenesis. Sequences affiliated with CH_4_-oxidizing archaeal Thermoplasmata displayed comparable abundance among the blue hole and the open sea waters, potentially explaining the low concentration of CH_4_ (<9 nmol l^−1^) in the oxic layers.

## Conclusions

The O_2_ deficiency is ongoing in global oceans, and understanding the biogeochemical responses to deogxygenation in various marine ecosystems will help our adaptation to such changes. The Sansha Yongle Blue Hole can act as an indicator of how O_2_ loss might influence microbially mediated biochemical processes in oligotrophic marine ecosystems.

O_2_ plays the most important role in affecting the microbial assemblages of the blue hole and surrounding open sea waters (44.7% of the total variation). The microbial composition occurring in oxic-to-suboxic zone has characteristic of that in the deep waters of surrounding open sea. That means, biochemical processes (e.g. NO_2_^−^ oxidation by *Nitrospina* and CH_4_ oxidation by archaean Marine Group II) in deep waters could occur in shallow waters when O_2_ is deficient. Moreover, heterotrophic aggregate-associated *Alteromonas* blooms and might enhance the NO_3_^−^ reduction process under O_2_ decrease, shifting the PNM towards the chlorophyll *a* peak. These all might influence carbon- and nitrogen-transforming reactions in the marine ecosystems.

The NO_3_^−^/NO_2_^−^-H_2_S transition zone sunstains a diverse microbial community capable of sulfur oxidation by denitrification in the blue hole, such as γ-, ε-Proteobacteria, and Chlorobi. These are ubiquitous in diverse suboxic marine environments. The depth-specific patterns and metabolic versatilities enable to prevent the escape of H_2_S produced from the bottom layer waters. On the other hand, low level of NO_2_^−^ and high level of H_2_S might limit anammox process, leading to NH_4_^+^ excessive.

## Methods

### Site locations, sampling, and biological analyses

Samples were collected in May 2017 aboard the R/V *Changhe Ocean*, a cargo ship, and an anchored working platform as previously described^20^. We established six sites in the Sansha Yongle Blue Hole and the surrounding waters: SYBL, C1, C2, C3, C4, and C5 (Fig. 1; Table 1). At each site, 5-L water samples were taken as described by Xie *et al*.^20^. All water samples were filtered through 0.22-µm acetate membranes using a vacuum pump while on board and then stored in liquid nitrogen for DNA extraction. A chlorophyll *a* fluorometer (Hydro-Bios Apparatebau GmbH, Kiel, Germany) was attached to a Conductivity Temperature Depth profiler (Sea-Bird SBE 911plus, Sea-Bird Electronics Inc., Bellevue, WA, USA) to measure chlorophyll *a*. Chlorophyll a was calculated from *in vivo* uncalibrated fluorescence. Prokaryotes were counted using a FACSCalibur flow cytometer (Becton Dickinson Biosciences, CA, USA) following the protocols of Marie *et al*.^84^.

We collected 40 water samples for DNA extraction across all six sites: SYBL0m, SYBL10m, SYBL30m, SYBL50m, SYBL70m, SYBL80m, SYBL90m, SYBL100m, SYBL125m, SYBL150m, SYBL200m, and SYBL300m; C3–0m, C3-10m, C3-30m, C3-50m, C3-75m, C3-100m, C3-150m, C3-200m, C3-300m; C4-0m, C4-10m, C4-30m, C4-50m, C4-75m, C4-100m, C4-150m, C4-200m, C4-300m; C1-0m, C1-10m; C2-0m, C2-10m, C2-30m; C5-0m, C5-10m, C5-30m. We sampled C4-300m (C4–300m-1) and SYBL125m (SYBL125m-1) repeatedly.

### DNA extraction, 16S rRNA polymerase chain reaction (PCR) amplification, and sequencing

Total genomic DNA was extracted from each sample using a FastDNA Spin Kit for Soil (MP Biomedicals, Santa Ana, CA, USA) following the manufacturer’s instructions. The concentration and quality (A260/A280 ratio) of each DNA sample were measured using a NanoDrop 2000 spectrophotometer (Thermo Scientific, Waltham, MA, USA).

The V3–V4 region of the 16S ribosomal RNA gene was PCR amplified using primers 341 F (CCTACGGGNGGCWGCAG)^85^ and 806 R (GGACTACHVGGGTATCTAAT)^86^; an eight-base barcode unique to each sample was added to each sequence. PCRs were performed in triplicate. Each 50-µl PCR contained 5 μl of 10 × KOD Buffer, 5 μl of 2.5 mmol 1^−1^ dNTPs, 1.5 μl of each primer (5 μ mol 1^−1^), 1 μl of KOD polymerase, and 100 ng of template DNA. The amplification cycling program was an initial denaturation at 95 °C for 2 min, followed by 27 cycles of denaturation at 98 °C for 10 s, annealing at 62 °C for 30 s, and extension at 68 °C for 30 s, with a final extension at 68 °C for 10 min. PCR products were purified using the AxyPrep DNA Gel Extraction Kit (Axygen Biosciences, Union City, CA, U.S.) following the manufacturer’s instructions. Equimolar volumes of purified amplicons were pooled and paired-end sequenced with an Illumina HiSeq. 2500 PE250 (Illumina, San Diego, CA, USA), following standard protocols, by GeneDenovo Biotechnology Co., Ltd. (Guangzhou, China).

Raw reads were deposited into the NCBI Sequence Read Archive (SRA) database (accession numbers: SAMN1036346–SAMN10363471).

### Bioinformatic analysis

Paired-end clean reads were merged as raw tags using FLSAH (v 1.2.11)^87^, with a minimum overlap of 10 bp and a mismatch error rate of 2%. We recovered 75044−109589 raw tags from each sample. Noisy sequences of raw tags were filtered using the QIIME (V1.9.1)^88^ pipeline with specific filtering conditions^89^ to obtain high-quality cleaned tags. All chimeric tags were removed. The remaining effective tags were clustered into OTUs with ≥ 97% similarity using the UPARSE pipeline^90^. The tag sequence with the highest abundance was selected as the representative sequence for each cluster. The representative sequences were assigned to organisms by a naive Bayesian model using the ribosomal database project classifier (Version 2.2)^91^, which is based on the SILVA database^92^. The abundances of major microbial divisions are shown as a percentage of total identifiable 16S rRNA gene sequences. Phylogenetic trees were constructed using the neighbor-joining algorithm implemented in MEGA4^93^. Bootstrapping was performed by resampling 1000 times. Bootstrap values <50% are not shown. The scale bars represent estimated changes per nucleotide.

### Metagenome sequencing and assembly

We used 1 μg DNA per sample (SYBL0m, SYBL30m, SYBL90m SYBL100m, SYBL150m, and SYBL300m) as input material for DNA library preparations. Sequencing libraries were generated using the NEBNext Ultra DNA Library Prep Kit for Illumina (New England Biolabs, Ipswich, MA, USA), following the manufacturer’s instructions. Index codes were added to attribute each sequence to a sample. The index-coded samples were clustered using a cBot Cluster Generation System (Illumina, San Diego, CA, USA), following the manufacturer’s instructions. After cluster generation, library preparations were sequenced on an Illumina HiSeq. 2500 platform (Illumina), and paired-end reads were generated. We recovered 66312588~87965958 clean reads from each sample. The Illumina sequencing data were assembled individually and by sample using MEGAHIT^94^ (the University of Hong Kong & L3 Bioinformatics Limited, Hong Kong, China; parameter:–k-min 21–k-max 81–k-step 20 -t 8). Overall, *de novo* assembly statistics were determined using BWA (Edition, 0.7.5a-r405)^95^, which calculated the percentage of paired or singleton reads realigned to the assembly. The unmapped reads from each sample were pooled and re-assembled using MEGAHIT to generate mixed assemblies. For each sample, the sample-derived and mixed assemblies were combined to obtain a final assembled contigs. A total of 2.1 Gb data were recovered from each sample.

### Gene prediction and cataloging

We predicted the open reading frames (ORFs) of the final contigs (>500 bp) using MetaGeneMark^96^. All predicted ORFs ≥300 bp in length were pooled, and ORFs more than ≥95% identical present in ≥90% of all reads were combined with CD-HIT^97^ in order to reduce the number of redundant genes in the downstream assembly step. Reads were realigned to predicted genes, and read numbers were counted using BWA. The final gene catalogue included only non-redundant genes with gene read counts >2. All unique ORFs were annotated against the Kyoto Encyclopedia of Genes and Genomes using DIAMOND^98^. Reads were filtered, and taxonomic profiles were generated based on cleaned reads with MetaOthello^99^.

Abundances of metabolic function genes were calculated relative to the putative single copy per organism of RNA polymerase subunit B (*rpoB*). Abundances per gene were normalized to gene length^4^.

$$ \% \,prokaryotic\,community=\frac{\frac{GeneA\,reads}{Length\,A}}{\frac{rpoB\,reads}{Length\,rpoB}}$$

### Statistical analysis

The similarity of the bacterial and archaeal composition across samples was analyzed by hierarchical clustering analysis in the “vegan” R package (R version 3.4.3)^100^. In this analysis, Hellinger distances for the relative abundances of phyla and genera among samples were calculated, coupled with the Ward linkage method. Statistically meaningful groups were then identified using fusion-level values and Mantle Pearson’s correlations in the “vegan” R package (R version 3.4.3)^100^. Redundancy analysis (RDA) was performed using Canoco 4.5 to assess the relationships between the biophysiochemical variables and microbial composition^101^. The significance of the variable was tested using Monte Carlo permutation tests with 499 unrestricted permutations (*P* < 0.05). Chlorophyll *a* and 14 physiochemical variables at the SYBL, C3 and C4 were standardized to *Z*–score values (zero mean, unit SD). These 14 physiochemical variables and methods were based on our parallel study^20^, and were shown in Table S1. The parameters included NO_2_^−^, NO_3_^−^, NH_4_^+^, SiO_3_^2−^, H_2_S, N_2_O, suspended particulate matter (SPM), CH_4_, dissolved organic carbon (DOC), particulate organic carbon (POC), temperature, salinity, particulate nitrogen (PN), and dissolved oxygen (DO).The Hellinger distances among the relative abundances of phyla were calculated for all samples. Pearson’s correlation analyses were carried out with SPSS statistics 17.0 software to test relationships among relative abundances of different microbial groups and environmental variables.

## Supplementary information

Supplementary information.
