# Supplementary material for: Microbial Diversity and Metabolic Potential in the Stratified Sansha Yongle Blue Hole in the South China Sea
Source: Sci Rep. 2020 Apr 6;10:5949. doi: 10.1038/s41598-020-62411-2 (PMC7136235; doi:10.1038/s41598-020-62411-2)
Supplement: Supplementary file 1 — Supplementary information. [file 41598_2020_62411_MOESM1_ESM.pdf]

Microbial Diversity and Metabolic Potential in the Stratified Sansha Yongle Blue Hole in the South China Sea

Peiqing He [1, 2, 3*], Linping Xie [1, 2], Xuelei Zhang [1, 2], Jiang Li [1, 3], Xuezheng Lin [1, 3], Xinming Pu [1, 2], Chao Yuan [1, 2], Ziwen Tian [4], Jie Li [5]

1. MNR Key Lab of Science and Technology for Marine Ecology and Environment, First Institute of Oceanography, Ministry of Natural Resources, 6 Xianxialing Road, Qingdao, 266061, P R China

2. Laboratory for Marine Ecology and Environmental Science, Qingdao National Laboratory for Marine Science and Technology, Qingdao 266071, China

3. Key laboratory of Natural Products of Qingdao, Qingdao, Shandong 266061, China

4. Research Center for Islands & Coastal Zone, First Institute of Oceanography, Ministry of Natural Resources, 6 Xianxialing Road, Qingdao, 266061, P R China

5. Marine Engineering Environment & Geomatic Center, First Institute of Oceanography, Ministry of Natural Resources, 6 Xianxialing Road, Qingdao, 266061, P R China

Running Head: microbial diversity and metabolic potential in a blue hole

Correspondence should be addressed to Peiqing He (Email: hepeiqing@fio.org.cn)

MNR Key Lab of Science and Technology for Marine Ecology and Environment, First Institute of Oceanography, Ministry of Natural Resources, Xianxialing Road, Qingdao, 266061, PR China. Tel: 86 53288969036. Fax: 86 53288967422

Table S1. Physiochemical variables and methods using redundancy analysis

Variables	Method
Silicate	Molybdosilicic acid method (Fanning and Pilson, 1973)
Nitrate, Nitrite	Standard pink azo dye method (Strickland and Parsons, 1972),
Ammonium	Hypobromite oxidation method (Strickland and Parsons, 1972).
Temperature, Salinity	A Conductivity Temperature Depth profiler (Sea-Bird SBE 911plus)
Suspended particulate matter	Gravimetric analysis
Dissolved organic carbon	High temperature catalytic oxidation using a Total Organic Carbon analyzer (Shimadzu, Japan) (Sugimura and Suzuki, 1988)
Particulate organic carbon, Particulate nitrogen	A CHN elemental analyzer (Elementar Analysensysteme GmbH, Germany).
Dissolved oxygen	Metrohm 848 Titrino plus sensor using the Winkler method (Metrohm, Switzerland)
Methane, nitrous oxide	Gas chromatography using gas-stripping methods (Zhang et al., 2004, 2006)
Sufide	Methylene blue method（State technology supervision bureau, 2007)

Physiochemical variables and methods were based on our parallel study[20].

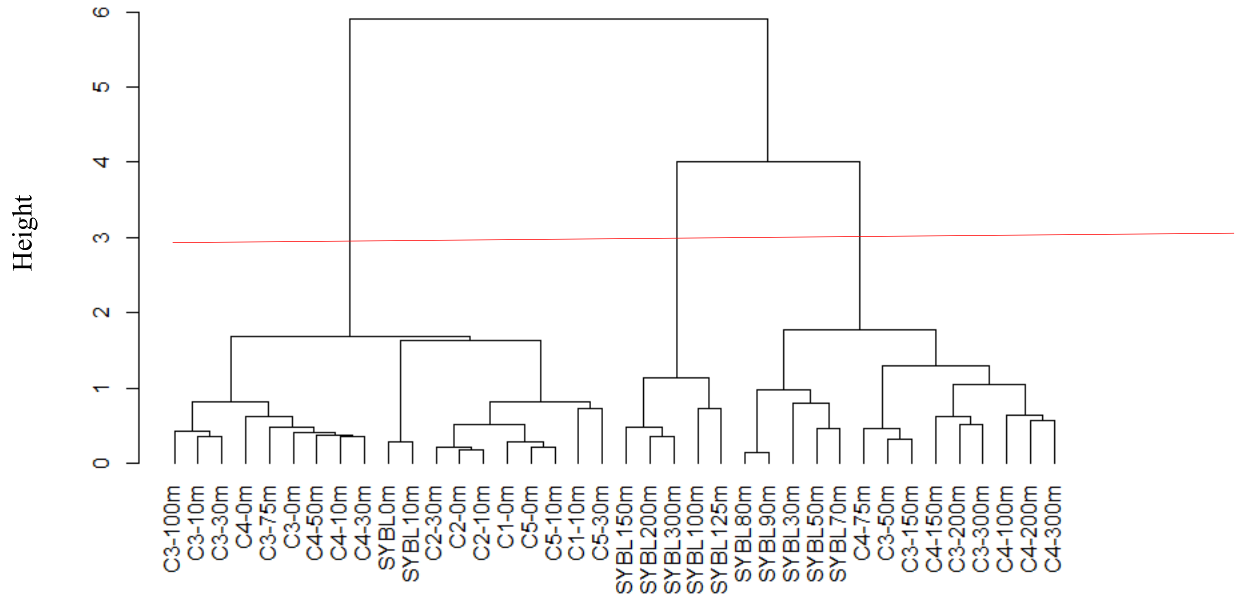

Figure S1. Cluster analysis of 1345 microbial genera from the Sansha Yongle Blue Hole and surrounding regions based on 16S rRNA amplicon sequences

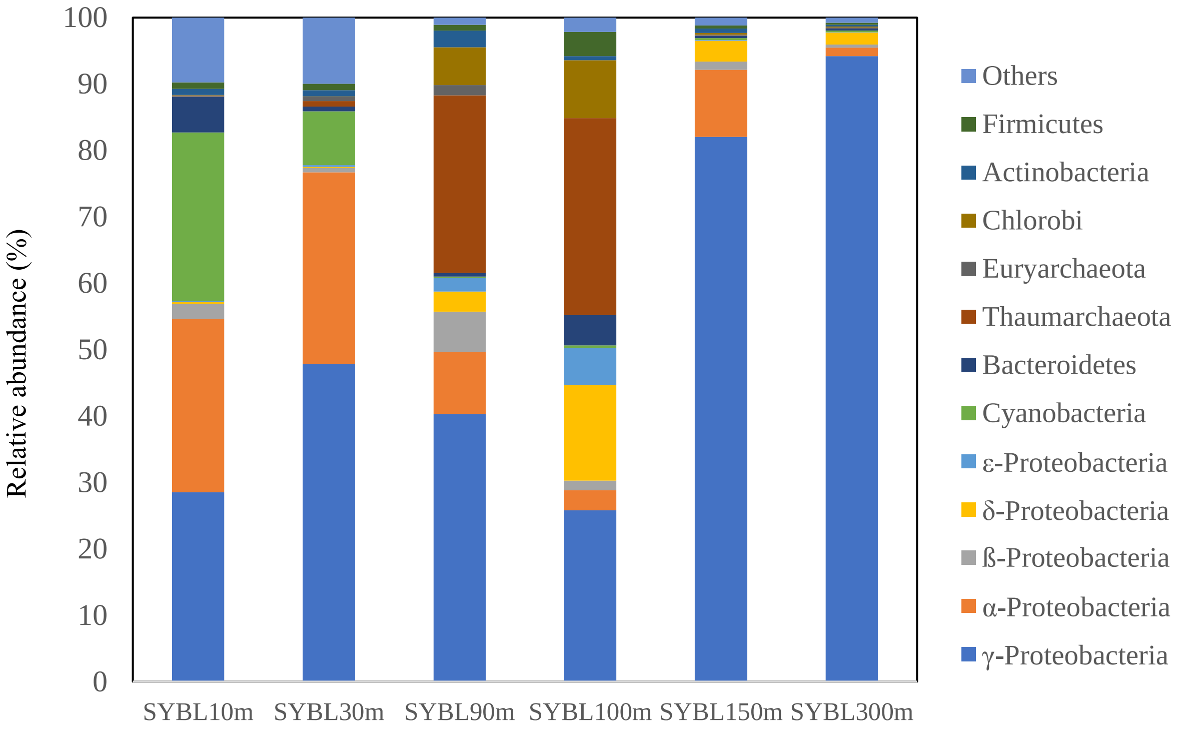

Figure S2. Relative abundance of microbial phyla from the Sansha Yongle Blue Hole based on metagenomic sequences

(a)

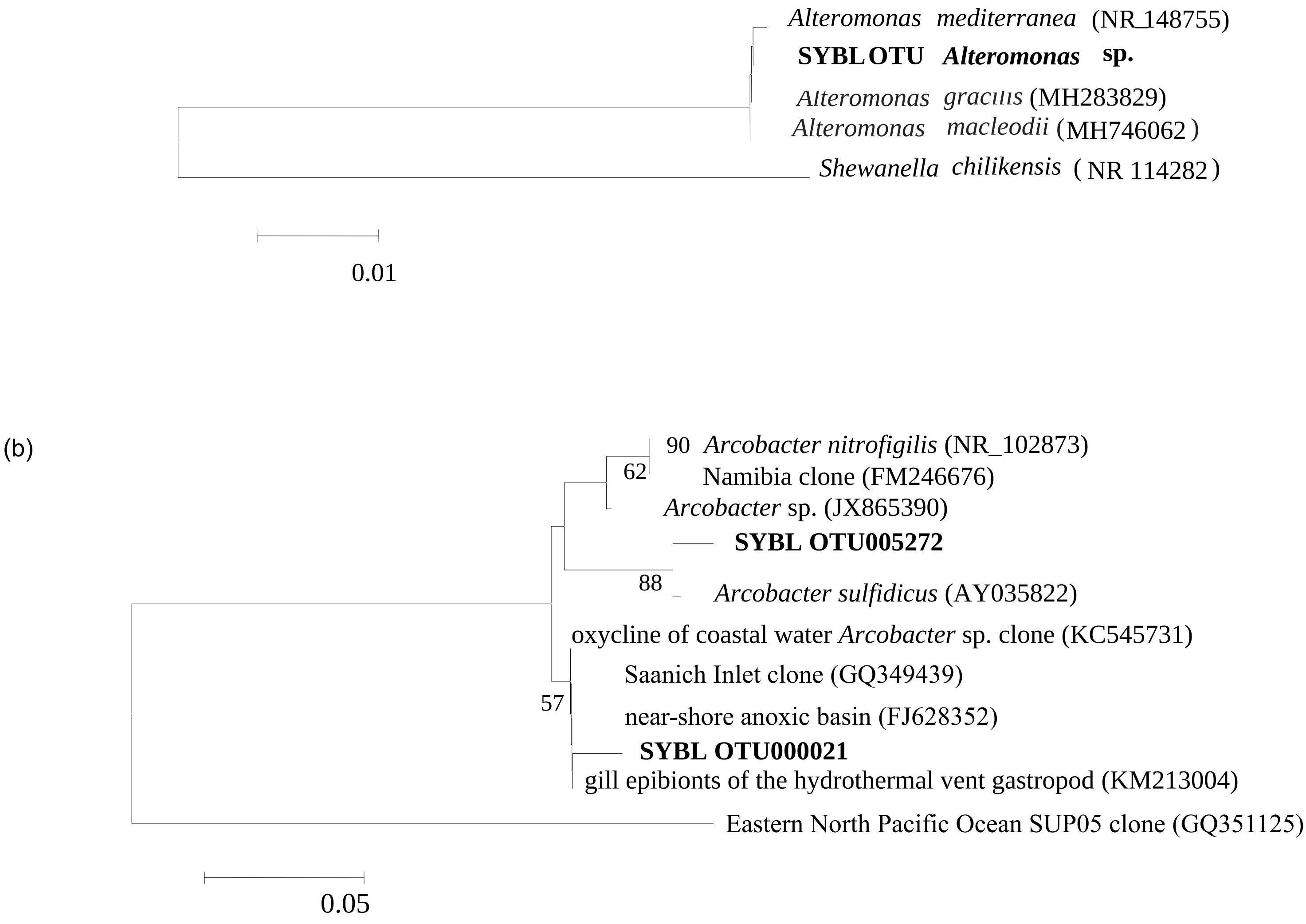

Figure S3. Phylogenetic trees of (a) nitrate-reducing bacteria and (b) sulfide-oxidizing bacteria, based on 16S rRNA sequences. Phylogenetic trees were constructed using the neighbor-joining (NJ) algorithm implemented in MEGA4[94]. Bootstrapping was performed by resampling 1000 times. Bootstrap values <50% are not shown.
